# Senescence Seclusion Syndrome: Appraising the Efficacy of Current Interventions

**DOI:** 10.7759/cureus.62684

**Published:** 2024-06-19

**Authors:** Noah Leton

**Affiliations:** 1 Department of Physiology, Neuroscience and Behavioral Sciences, St. George's University, St. George's, GRD

**Keywords:** intervention efficacy, intervention assessment, social isolation in the elderly, loneliness in the elderly, senescence seclusion syndrome

## Abstract

The proportion of senior citizens in the global population has been on a steady rise, and the current population is forecasted to double in a few decades. Against this backdrop, the prevalence of senescence seclusion syndrome, characterized by prolonged social isolation in the elderly, is increasing concurrently. Given the profound threats this syndrome poses to the mental, physical, and social well-being of this vulnerable demographic, implementing effective interventions is imperative to mitigate these threats and enhance the quality of life. This review aims to assess the efficacy of the interventional modalities critically.

Studies were reviewed following comprehensive searches in databases such as PubMed, Scopus, and Google Scholar, and focusing on empirical studies from 2019 to 2024 that evaluated the efficacy of the major intervention categories - social, technological, psychological, and physical interventions.

The findings indicate that initiatives that promote consistent social engagement, such as community-organized social events and structured group activities, significantly reduced loneliness and bolstered social connections. Furthermore, technological interventions, including artificial intelligence and virtual reality, have notably enhanced elderly connectivity with their communities. Additionally, psychological interventions, such as cognitive-behavioral therapy, have also been effective in alleviating symptoms of anxiety and depression associated with the syndrome, with group sessions enhancing social interaction and significantly diminishing isolation. Moreover, physical interventions involving group exercises and other activities have fostered improvements in the physical, mental, and social well-being of the elderly.

This study underscores the importance of a multifaceted approach that is individualized according to preference and circumstance in addressing senescence seclusion syndrome.

## Introduction and background

"I do not want to live past 60 years because I do not want to get old and become a burden to family and society. But most of all, I do not want to be alone”. These were the exact words of a former colleague several years ago. Opinions will certainly differ, but an inclination toward longevity is more likely. Old age is undoubtedly golden, and the elderly contribute in no little way to everyday family life and society. The aging of an individual or a group of individuals in a population reflects several factors such as socioeconomic status, healthcare availability, affordability and accessibility, genetics, and environmental factors [1].

The phenomenon of aging and accompanying challenges

Aging is an inexorable biological phenomenon characterized by a gradual deterioration in physiological capabilities, culminating in heightened susceptibility to illness, disability, and death [2]. This progression impacts various facets of life, including health, social interactions, and mental well-being [1]. In 2021, 1 in 10 people worldwide were aged 65 or older [1]. With an inclining global demographic, by 2050, it is projected that this age group will make up one in six people globally [1]. Consequently, the accompanying morbidities present a plethora of challenges for medical, public health, and social care infrastructures. These challenges which have a wide spectrum and are far-reaching have both a direct and indirect impact on the individual and the society. A seemingly inconspicuous, but equally devastating adversity encountered by the elderly is senescence seclusion syndrome (SSS).

An insight into SSS

SSS describes a state where older adults endure profound social isolation and withdrawal [1]. This condition transcends the mere absence of social interactions to include the subjective sense of isolation, a significant forecaster of diminished life quality, and various negative health consequences [2]. Affected individuals often retreat from social activities, curtail their community involvement, and frequently perceive themselves as overlooked by society, and with the presence of events typically associated with aging, such as the loss of a spouse, retirement, the onset of disability, or children leaving home (the so-called empty nest syndrome), this syndrome can worsen [3].

The significance of SSS in the context of aging populations is considerable. As individuals age, they face an increased risk of encountering isolation factors, including bereavement, chronic illnesses, and sensory impairments such as hearing loss [3]. These elements, alongside the dwindling of social networks due to the deaths of peers and relatives, intensify feelings of loneliness and isolation. Also, cultural and contextual aspects of aging play a role in the severity of this syndrome. The repercussions of SSS are extensive, impacting mental health by heightening the likelihood of depression and anxiety, affecting physical health by increasing risks of cardiovascular diseases and diminished immune function, and even impairing cognitive functions, potentially hastening the onset and progression of dementia [3].

Addressing SSS: significance and consequences

Addressing SSS is imperative for several reasons, which span psychological well-being, physical health, quality of life, and economic impact [2,3]. Regarding psychological well-being, robust social connections are fundamental to maintaining mental health. They provide critical emotional support, help in coping with stress, and play a significant role in reducing the prevalence of mental health disorders such as depression, anxiety, and cognitive decline [2,3]. Concerning physical health, regular interaction with peers and communities can boost motivation for physical activity, which is vital for maintaining fitness and managing health [2,3]. Socially active individuals are often more engaged in health-promoting behaviors, which can significantly diminish the risk of developing chronic diseases such as heart disease, diabetes, and obesity [3]. About quality of life, meaningful social interactions can profoundly enrich one's life experiences. They contribute to a sense of belonging and purpose, enhance emotional resilience, and promote overall happiness and life satisfaction [3]. In terms of economic impact, addressing social isolation not only improves individual health outcomes but also has broad economic benefits [3]. By reducing the extent of loneliness, societies can decrease the associated healthcare costs. This reduction comes from less frequent medical visits and lower incidences of mental and physical health issues that require intervention, ultimately easing the financial burden on healthcare systems [3].

Failure to tackle SSS predisposes to consequences, some of which may be dire. These include deteriorating mental health. In this case, loneliness is significantly associated with increased risks of depression, anxiety, and cognitive decline. The Centers for Disease Control and Prevention (CDC) report that more than 20% of adults over the age of 50 experience persistent loneliness, which substantially affects their mental and physical well-being [2]. A decline in physical health is another repercussion. Social isolation correlates with heightened susceptibility to several physical health issues, such as elevated blood pressure, heart disease, and compromised immune function. According to the CDC, isolated elderly individuals face a 50% increased risk of developing dementia [2]. The magnitude of SSS was described in a study which indicated that insufficient social ties can adversely impact health to an extent comparable to smoking up to 15 cigarettes daily [3].

Furthermore, elevated mortality rates have been observed, which result from the combined psychological and physical impacts of loneliness [2,3]. Studies have demonstrated that elderly people experiencing social isolation had a 29% increased mortality rate compared to those maintaining regular social interactions [2,3]. Also, social isolation markedly raises the likelihood of premature death from all causes, a risk comparable to that posed by smoking, obesity, and physical inactivity [3]. Another study revealed that elderly individuals who are socially isolated face a 25-30% increased risk of early mortality [4]. Addressing social isolation in elderly populations is a critical priority. Implementing effective interventions can markedly improve the health and well-being of this at-risk group, thereby reducing mortality rates, enhancing overall quality of life, and creating healthier, more inclusive communities.

Overview of interventions

The interventions are broadly grouped into social, technological, psychological (psychological-based), and physical [3]. Other interventions such as spiritual approaches are also being utilized. Social interventions aim to bolster social ties through community-based groups, consistent social activities, and volunteer efforts that promote active participation among the elderly [3]. Notable examples include senior centers, planned group excursions, and social clubs that foster ongoing engagement and communal unity. Technological initiatives incorporate the use of social media, video conferencing, and tailor-made applications that enable seniors to maintain connections with family, friends, and peers from a distance [3]. Additionally, virtual reality (VR) experiences offer simulated settings that provide both social interaction and cognitive stimulation. Psychological methods concentrate on the psychological elements of isolation [3]. They encompass counseling services, cognitive-behavioral therapies (CBTs), and group therapy sessions aimed at addressing feelings of loneliness and their psychological effects. Programs focusing on mindfulness and stress management are also employed to enhance mental well-being. Lastly, as regards physical interventions, physical activity is vital for the health of the elderly, addressing both social and physiological needs. Activities such as group fitness classes, dance sessions, and walking clubs do more than keep seniors physically active; they also create avenues for social interaction and community involvement [3].

The efficacy of these interventions can differ greatly depending on a range of factors, such as the individual's health condition, personal choices, socio-economic background, and the caliber of implementation [3]. For instance, although technological interventions might benefit some, they may not be appropriate for those with limited tech skills or access. Likewise, physical interventions could be impractical for individuals with substantial mobility challenges [3].

Given the variability in outcomes of these interventions, it is imperative to undertake an exhaustive review to determine their overall efficacy and pinpoint which strategies are most advantageous for particular groups within the elderly population. Conducting such reviews can aid in refining intervention strategies, ensuring they are both effective and tailored to meet the varied needs of the elderly. This is crucial for the expansion of successful programs and for making well-informed choices regarding the allocation of resources and the development of policies aimed at mitigating isolation among the elderly.

Addressing research gaps

The corpus of research dedicated to mitigating isolation among the elderly has grown significantly in recent years. Despite this growth, critical deficiencies persist, particularly in assessing the efficacy of various interventions and their enduring impacts. Bridging these gaps is vital for crafting effective strategies that alleviate immediate loneliness and promote long-term enhancements in the well-being of elderly communities.

Lack of Comprehensive Comparative Studies

Numerous studies examine specific interventions such as social, technological, psychological, or physical. However, there is a noticeable deficiency in thorough comparative analyses that gauge the effectiveness across these modalities [3]. This gap hinders policymakers and healthcare professionals in identifying the most beneficial interventions for distinct segments of the elderly demographic.

Neglect of Long-Term Consequences

While many investigations focus on the immediate outcomes of interventions, fewer probe their extended effects. Grasping these prolonged impacts is crucial for evaluating true effectiveness. Extended follow-up studies are imperative to ascertain whether initial gains, like diminished loneliness or enhanced mental health, are sustained over time [3].

Inconsistencies in Intervention Deployment

There is substantial variation in how interventions are applied across different contexts, influencing their success. Yet, the literature often overlooks these discrepancies and fails to dissect which elements of implementation are most crucial for achieving positive outcomes [3].

Exclusion of Diverse Populations

Existing research frequently ignores the cultural, socioeconomic, and geographical diversity within elderly groups. Diverse populations might react differently to the same interventions due to varying cultural norms or accessibility issues [3]. This oversight leads to a generalized approach in recommendations, which may not be effective for all groups.

Justification of the study

A detailed literature review that amalgamates recent findings is essential to address these deficiencies for several reasons [1-3]:

Holistic Perspective

Such a review can amalgamate data from multiple studies to offer a comprehensive perspective on the effectiveness and long-term impacts of interventions. This integrated approach helps to better understand which strategies are most advantageous and under which conditions.

Evidence-Based Recommendations

By reviewing a wide array of studies, a literature review can generate precise, evidence-based recommendations tailored to various elderly groups and contexts. This is critical for developing specific interventions that can be customized to meet unique needs.

Foundation for Future Studies

A literature review that pinpoints existing research gaps and inconsistencies can guide future research directions. It can illuminate areas requiring further exploration, especially regarding long-term outcomes and the adaptability of interventions to different demographic groups.

Implications for Policy and Practice

Insights from a thorough literature review can influence policy decisions and the design of healthcare practices by providing well-substantiated recommendations on the most effective and sustainable interventions.

All in all, a literature review would not only deepen our understanding of SSS but also steer healthcare professionals, policymakers, and scholars toward developing more precise and enduring solutions to tackle the complexities of this condition.

Aim of the study

This review aims to (1) assess the effectiveness of interventions, (2) analyze long-term impacts, (3) guide future research, and (4) inform policy and practice.

## Review

This review focuses on studies published from 2019 to 2024. This period guarantees that the review includes the latest research, capturing current developments and breakthroughs in interventions for elderly isolation. Comprehensive searches were carried out on databases such as PubMed, Scopus, and Google Scholar, utilizing the following search query: senescence seclusion syndrome OR elderly isolation OR senescence seclusion OR geriatric isolation OR loneliness in the elderly AND, interventions. Primary studies on interventions and outcomes are included in this study. Exclusion criteria include studies published before 2019, studies on interventions that do not directly address SSS, non-interventional studies, and studies on younger adults. A total of 40 studies were selected and reviewed.

The review is structured primarily by the type of intervention, further detailing the features, effectiveness, and outcomes of these interventions. It also involves a comparative analysis to determine the most effective in particular circumstances, as well as a description of policy and practical implications.

Social interventions

Social interventions aim to enhance social connections and mitigate feelings of isolation among older adults [5]. These initiatives create opportunities for meaningful interactions, foster community engagement, and provide support networks [5-11].

Community and Group-Based Programs

Community and group-based programs are among the most common social interventions targeting loneliness in older adults [5,6]. These programs facilitate regular social interactions through organized activities, group outings, and communal spaces where older adults can meet and engage with peers. Lai et al. examined the impact of peer-based interventions on loneliness and social isolation among older Chinese immigrants in Canada [5]. The randomized controlled trial (RCT) demonstrated that peer support groups significantly reduced feelings of loneliness and enhanced social engagement. These findings suggest that culturally tailored peer support interventions can be particularly effective in addressing the unique social needs of immigrant populations. Similarly, Rodríguez-Romero et al. conducted a study that involved social programs to reduce perceived loneliness among community-dwelling older adults [6]. The study found significant reductions in loneliness levels, highlighting the effectiveness of such programs in fostering social connections and improving overall well-being.

Volunteering Programs

Another effective social intervention is volunteering, which provides older adults with opportunities to engage in meaningful activities and build social connections [7]. Volunteering not only alleviates loneliness but also boosts a sense of purpose and belonging. In a dual RCT conducted in 2024, Warner et al. evaluated the effects of volunteering on loneliness and social and mental health outcomes among older adults [7]. The study found that those who participated in volunteering activities for six months showed significant reductions in loneliness and improvements in mental health. These results emphasize the significance of volunteer programs in enhancing social well-being and psychological health among the elderly.

Creative and Cultural Activities

Creative and cultural activities have been shown to positively impact social well-being and cognitive health [8,9]. Activities such as singing, dancing, and participating in arts and crafts provide older adults with opportunities for social interaction, self-expression, and mental stimulation [8,9]. A study exploring a singing group program on the subjective and social well-being of older adults demonstrated that participation in the singing group significantly enhanced both subjective well-being and social connections [8]. The study underscored the therapeutic potential of group singing in reducing loneliness and fostering emotional health. In like fashion, Watson et al. researched the benefits of creative aging programs, which included various artistic and cultural activities, on the well-being of Canadian seniors [9]. The intervention study found that engaging in creative activities promoted healthy aging and improved quality of life. These findings indicate that creative programs can serve as effective interventions for boosting social engagement and overall well-being.

Peer Companionship and Support Groups

Peer companionship programs provide older adults with regular social interactions through matched companionship [10,11]. These programs often involve trained volunteers or peers who regularly visit or interact with older adults, offering emotional support and companionship [10,11]. In 2021, a pragmatic, nonblinded, parallel-group RCT was carried out to evaluate the effects of peer companionship on the mental health of older adults in primary care settings [10]. The study discovered that peer companionship significantly improved mental health outcomes, including reductions in depression and anxiety, and enhanced social connectedness. These findings demonstrate the effectiveness of peer companionship in addressing social and psychological needs. The effect of support groups on retirement syndrome in older adults was further emphasized by showing that participation in support groups significantly reduced symptoms of retirement syndrome and feelings of loneliness [11]. This underscores the importance of structured support groups in aiding older adults to navigate life transitions and maintain social connections.

Effectiveness of Social Interventions and Best Practices

The reviewed studies consistently show that social interventions are effective in reducing loneliness and enhancing the quality of life for older adults [5-11]. Key factors contributing to the success of these interventions include (1) regular and structured interactions - programs that offer consistent and structured social interactions tend to be more effective in reducing loneliness; (2) cultural relevance - interventions that are culturally tailored to meet the specific needs of diverse populations show greater efficacy; (3) meaningful engagement - activities that provide a sense of purpose and meaningful engagement, such as volunteering and creative pursuits, are particularly beneficial; and (4) support networks - providing access to peer support and companionship can address both social and emotional needs, offering comprehensive support [5-11].

Gaps and Future Directions

Despite the positive outcomes, there are areas where further research is needed, such as (1) long-term impact - more studies are needed to evaluate the long-term sustainability of social interventions; (2) scalability - research should explore how these interventions can be scaled and adapted to different community settings; and (3) diverse populations - future studies should focus on the effectiveness of interventions across various cultural, socio-economic, and geographic contexts [5-11].

Indeed, social interventions play a critical role in addressing loneliness among older adults. By facilitating social engagement, providing emotional support, and promoting meaningful activities, these interventions significantly enhance the well-being and quality of life of the elderly. Continued research and policy support are essential to expand and refine these interventions, ensuring they are accessible and effective for all older adults [5-11].

Technological interventions

Technological interventions have emerged as promising solutions to address social isolation and loneliness among older adults. These interventions leverage digital tools and platforms to facilitate social interaction, provide mental health support, and enhance overall well-being [12,13]. This section reviews various technological interventions, their implementation, and their effectiveness based on recent studies.

Digital and Online Platforms

Digital and online platforms have become increasingly popular for connecting older adults with their communities and loved ones. These platforms include social media, video conferencing tools, and online communities designed specifically for the elderly [12,13]. Following an investigation of the effectiveness of online interactive courses during the COVID-19 pandemic, it has been demonstrated that these courses significantly reduced loneliness and improved the quality of life for older adults [12]. This highlights the potential of digital platforms to provide social interaction and mental stimulation, particularly during periods of physical isolation. Similarly, Tsai et al. explored the effects of a smartphone-based videoconferencing program for older nursing home residents [13]. The quasi-experimental study demonstrated that the program significantly reduced depression and loneliness while improving the quality of life. These findings suggest that easy-to-use videoconferencing tools can effectively connect nursing home residents with their families and peers, mitigating feelings of isolation.

Mobile Health (mHealth) and CBT

mHealth applications and CBT delivered through digital platforms offer innovative approaches to providing mental health support to older adults [14,15]. During an evaluation of a low-intensity CBT intervention supported by mHealth to reduce loneliness among older people, it was demonstrated that the mHealth-supported CBT intervention was effective in decreasing feelings of loneliness [14]. This suggests that mobile applications can be a viable tool for delivering therapeutic interventions to the elderly, offering flexibility and accessibility. Also, as regards the effect of an online conversational skills coach on social engagement among older adults, it has been revealed that the intervention improved social engagement and reduced loneliness [15]. This highlights the potential of digital coaching programs to enhance communication skills and foster social connections.

Telehealth and Tele-Delivered Interventions

Telehealth and tele-delivered interventions have become crucial, especially during the COVID-19 pandemic, for providing healthcare and social support to older adults [12]. In an RCT comparing tele-delivered behavioral activation with tele-delivered friendly visits for homebound older adults, it was shown that tele-delivered behavioral activation significantly improved social connectedness compared to friendly visits [16]. This indicates that structured psychological interventions delivered via telehealth can be more effective in addressing loneliness than simple social interactions. By examining the one-year impact of tele-delivered behavioral activation versus friendly visits on social connectedness, there were sustained improvements in social connectedness for those who received behavioral activation, demonstrating the long-term benefits of tele-delivered psychological interventions [17].

Virtual Reality (VR) and Augmented Reality (AR)

VR and AR technologies are being explored for their potential to create immersive experiences that can reduce feelings of loneliness and improve mental health among older adults [18]. The Social and Cognitive Online Training (SCOT) project, a digital RCT aimed at promoting socio-cognitive well-being in older adults, was introduced in 2024, demonstrating that VR-based training significantly improved socio-cognitive well-being [18]. This suggests that immersive technologies can provide engaging and therapeutic experiences for the elderly.

Effectiveness of Technological Interventions and Best Practices

The reviewed studies confirm the effectiveness of technological interventions in tackling SSS [12-18]. Key factors contributing to the success of these interventions include (1) accessibility and usability - technologies that are easy to use and accessible to older adults, such as smartphones and tablets, are more likely to be adopted and effective; (2) engaging content - interactive and engaging content, such as online courses and VR experiences, can provide mental stimulation and foster social connections; (3) personalization - personalized interventions, such as digital coaching and tailored therapeutic programs, can address the specific needs of older adults, enhancing their effectiveness; (4) support and training - providing adequate support and training for older adults to use these technologies is crucial for their successful implementation and sustained use [12-18].

Gaps and Future Directions

Regarding technological interventions, more light needs to be shed on the following areas: (1) digital literacy - research should explore ways to improve digital literacy among older adults to ensure they can effectively use these technologies; (2) long-term impact - more studies are needed to assess the long-term sustainability and effectiveness of technological interventions; (3) equity and accessibility - future research should focus on making these technologies accessible to diverse populations, including those with limited resources or technological skills; (4) integration with other interventions - exploring the integration of technological interventions with other social, psychological, and physical interventions can provide a more holistic approach to addressing loneliness in older adults [12-18].

All in all, technological interventions offer innovative and effective solutions for reducing loneliness and improving the well-being of older adults. By leveraging digital platforms, mHealth applications, telehealth, and immersive technologies, these interventions can provide accessible, engaging, and personalized support. Continued research and development are essential to enhance their effectiveness, ensure equitable access, and integrate them into comprehensive care strategies for the elderly [12-18].

Psychological interventions

Psychological interventions focus on addressing the psychological and emotional aspects of loneliness among older adults and often involve counseling, psychotherapy, group therapy, and other structured psychological support methods [19-22]. The goal is to improve mental health, enhance social connections, and provide coping strategies for dealing with feelings of isolation [19-22]. This section reviews various therapeutic interventions, their implementation, and their effectiveness based on recent studies.

CBT

CBT is a well-established psychological intervention that helps individuals identify and modify negative thought patterns and behaviors and has been effectively adapted for older adults to address loneliness and associated mental health issues [19]. CBT encompasses various forms of therapy, including mindfulness therapy. Regarding CBT, the effectiveness of group CBT in reducing loneliness and improving social connections among a community sample of older adults yielded significant results [19]. These results highlight the potential of CBT in addressing the cognitive and behavioral components of loneliness. Recall the effectiveness of the mHealth-supported CBT intervention in decreasing feelings of loneliness [14].

Psychotherapeutic Interventions

Psychotherapeutic interventions encompass a range of therapies, including individual counseling, group therapy, and psychodynamic therapy, aimed at addressing deep-seated emotional issues and promoting mental well-being [20]. On the institution of psychotherapeutic interventions for homebound individuals, significant improvements in psychological well-being have been reported, indicating that targeted psychotherapeutic support can effectively reduce loneliness and enhance mental health [20].

Resilience and Wisdom-Focused Interventions

Interventions that focus on building resilience and cultivating wisdom have shown promise in enhancing the mental health and social well-being of older adults, and aim to develop coping strategies, foster a positive outlook, and enhance emotional regulation [21]. In a pilot-controlled clinical trial conducted to evaluate a remotely administered resilience- and wisdom-focused intervention, it was revealed that the intervention reduced perceived stress and loneliness among older adults, suggesting that fostering resilience and wisdom can be effective strategies for combating loneliness [21].

Behavioral Activation Therapy

Behavioral activation therapy is a psychological approach that encourages individuals to engage in meaningful activities to improve their mood and overall well-being and is particularly effective for older adults experiencing depression and loneliness [22]. The impact of engaging in socially and interpersonally rewarding activities as part of behavioral activation therapy for late-life depression has been investigated, with the revelation that engagement in these activities predicted better outcomes in reducing depression and loneliness [22], highlighting the importance of encouraging positive behavioral changes. As earlier indicated, tele-delivered behavioral activation significantly improved social connectedness compared to friendly visits, indicating the effectiveness of structured psychological interventions delivered via telehealth [16].

Group Therapy and Support Groups

Group therapy and support groups provide a platform for older adults to share their experiences, receive emotional support, and build social connections, while creating a sense of community and reducing feelings of isolation [10,11]. The impact of peer companionship on the mental health of older adults in primary care settings has been assessed, with significant improvement in mental health outcomes, including reductions in depression and anxiety, as well as enhanced social connectedness [10]. As earlier mentioned, support groups significantly reduce symptoms of retirement syndrome and feelings of loneliness, underscoring the importance of providing structured group support to help older adults navigate life transitions [11].

*Effectiveness of Psychological* *Interventions and Best Practices*

Psychological interventions have been demonstrated to be impactful in reducing loneliness and enhancing the mental health and social well-being of older adults [10,11,19-22]. Central elements that enhance success include (1) structured approaches - interventions that follow a structured psychological approach, such as CBT and behavioral activation therapy, show significant effectiveness in reducing loneliness; (2) group dynamics - group therapy and support groups provide social support and a sense of community, which are crucial for alleviating loneliness; (3) remote delivery - telehealth and remotely delivered interventions, such as teletherapy and online support groups, offer accessible options for older adults, particularly those who are homebound or have mobility issues; (4) focus on resilience - interventions that build resilience and emotional regulation skills help older adults cope better with loneliness and stress [10,11,19-22].

Gaps and Future Directions

These include (1) long-term impact - more studies are needed to assess the long-term sustainability and effectiveness of psychological interventions; (2) diverse populations - research should explore the adaptability and effectiveness of psychological interventions across diverse cultural and socio-economic contexts; (3) combination with other interventions - exploring the integration of psychological interventions with social, technological, and physical approaches can provide a comprehensive strategy for addressing loneliness in older adults; (4) barriers to access - identifying and addressing barriers to accessing psychological interventions, such as stigma, cost, and availability, is crucial for their success [19-22].

To conclude, psychological interventions offer significant benefits for reducing SSS and improving the mental health and overall well-being of older adults. By leveraging structured psychological approaches, group support, and resilience-building strategies, these interventions can effectively address the emotional and psychological aspects of loneliness [19-22]. Continued research and development are essential to optimize these interventions, ensure their accessibility, and integrate them into holistic care strategies for the elderly [19-22].

Physical interventions

Physical interventions aimed at reducing loneliness among older adults focus on promoting physical activity, enhancing physical health, and fostering social interaction through group-based and community-based activities [23-27]. These interventions, which include aerobic exercise, strength training, flexibility and balance training, functional training, occupational therapy, physiotherapy, etc., leverage the benefits of physical exercise to improve mental health, reduce feelings of isolation, and increase overall well-being [23-27]. This section reviews various physical interventions, their implementation, and their effectiveness based on recent studies.

Community-Based Exercise Programs

Community-based exercise programs are designed to promote physical activity among older adults while providing opportunities for social interaction and often include group exercises, fitness classes, and organized physical activities that encourage participants to engage with each other [23]. Following an RCT to evaluate the effectiveness of a community-based exercise program enhanced by a self-management component, it was shown that this program significantly improved independent living among older adults [23]. Participants reported enhanced physical health and increased social engagement, demonstrating the dual benefits of physical activity and social interaction.

Group Physical Activities

Engaging in group physical activities, such as dance classes, yoga sessions, and walking clubs, provides older adults with both physical and social benefits, which not only help maintain physical fitness but also create a sense of community and belonging [24,25]. Zengin et al. investigated the impact of synchronous tele-exercise on physical fitness, quality of life, and mood in older people. The study showed that tele-exercise maintained physical fitness and improved quality of life and mood [24]. This suggests that group-based physical activities, even when delivered remotely, can effectively reduce feelings of isolation, and promote well-being. Similarly, Liu et al. explored the effectiveness of a reminiscence therapy-based hybrid board game, which combines physical and cognitive activities [25]. The study found that the intervention significantly decreased anxiety and loneliness levels in older adults. The hybrid approach provided a holistic intervention, integrating physical exercise with cognitive and social stimulation [25].

Structured Exercise Programs

Structured exercise programs, such as aerobic exercises, strength training, and balance exercises, are tailored to meet the physical health needs of older adults often under the supervision of fitness instructors or healthcare professionals to ensure safety and effectiveness [26]. The MIPAM trial, a 12-week intervention using motivational interviewing and physical activity monitoring (MIPAM) to enhance daily physical activity in community-dwelling older adults, has been described [26]. Although the study protocol primarily focuses on physical activity, it also highlights the potential for improving social engagement through structured exercise programs [26].

Physical Activity Combined With Social Support

Combining physical activity with social support can enhance the effectiveness of interventions aimed at reducing loneliness, and include elements such as peer support, group discussions, and social events alongside physical exercises [27]. Regarding evaluating the effects of participatory group-based care management on the well-being of older people living alone, it has been revealed that the intervention significantly improved well-being by combining physical activity with social support [27], highlighting the importance of integrating social elements into physical interventions.

Effectiveness of Physical Interventions and Best Practices

The effectiveness of physical interventions in mitigating SSS has been demonstrated in several studies [23-27]. For improved success rates, however, the following vital elements should be considered: (1) group participation - physical activities that involve group participation foster social connections and create a sense of community; (2) structured programs - well-structured exercise programs, tailored to the needs of older adults, ensure safety and effectiveness; (3) combination with social support - integrating social support elements into physical activity programs enhances their impact on reducing loneliness; (4) remote accessibility - tele-exercise and remote physical activities can be effective alternatives, particularly during periods of physical distancing, such as during the COVID-19 pandemic [12,23-27].

Gaps and Future Directions

While physical interventions show promise, several areas require further research. These are (1) long-term impact - more studies are needed to assess the long-term sustainability and effectiveness of physical interventions; (2) diverse populations - research should explore the adaptability and effectiveness of physical interventions across diverse cultural and socio-economic contexts; (3) integration with other interventions - exploring the integration of physical interventions with technological and psychological approaches can provide a comprehensive strategy for addressing loneliness in older adults; (4) barriers to participation - identifying and addressing barriers to participation, such as mobility issues, transportation challenges, and accessibility, is crucial for the success of these interventions [23-27].

To conclude, physical interventions, by promoting physical activity through community-based programs, group activities, and structured exercise, not only enhance physical health but also foster social connections [23-27]. Continued research and development are essential to optimize these interventions, ensure their accessibility, and integrate them into holistic care strategies for the elderly [23-27].

Comparative analysis of interventions

To fully grasp the range of interventions for reducing SSS, it's essential to conduct a comparative analysis to pinpoint the most effective approaches. This synthesis evaluates social, technological, psychological, and physical interventions, scrutinizing their strengths, limitations, and overall effectiveness. By amalgamating findings from these intervention types, this analysis offers a comprehensive perspective on strategies to tackle loneliness and enhance the well-being of older adults.

Social Interventions

Strengths include (1) community and group-based programs - these interventions foster direct social interactions and community engagement, creating a sense of belonging and support. Studies demonstrate significant reductions in loneliness and improved social connectedness [5,6]. The effectiveness of these programs is enhanced by regular, structured interactions and culturally relevant programs; (2) Volunteering programs - these programs provide older adults with meaningful activities and a sense of purpose, with studies showing significant improvements in social and mental health outcomes [7]. In this case, structured opportunities and support from organizations are critical for success. The main limitations to social interventions are accessibility and sustainability. Regarding accessibility, some older adults may face barriers to participation due to mobility issues or lack of transportation. For sustainability, the long-term sustainability of these programs can be challenging without continuous funding and support [5-11].

Technological Interventions

Notable strengths of technological interventions are (1) digital and online platforms - these offer scalable and accessible solutions for social interaction and mental stimulation. The effectiveness of these platforms was confirmed with a demonstration of reduced loneliness and improved quality of life through online courses and videoconferencing [12,13]. However, ease of use and engaging content are crucial for adoption and effectiveness; (2) mHealth and CBT applications - these provide flexible and accessible mental health support effectively while reducing loneliness [14,15]. Personalized, user-friendly interfaces even further enhance effectiveness. The main drawbacks are digital literacy, in which older adults with limited technological skills may struggle to use these platforms, and access to technology, which stems from the fact that not all older adults have access to the necessary devices and internet connectivity [12-18].

Psychological Interventions

Strengths include (1) CBT, which is effective in addressing cognitive and behavioral aspects of loneliness [19]. In this case, however, structured psychological approaches and professional facilitation are important. (2) Psychotherapeutic and resilience-focused interventions - these address deep-seated emotional issues, build coping strategies, and show improvements in psychological well-being and reduced loneliness [20,21]. Tailored interventions that focus on resilience and emotional regulation enhance the effectiveness of these interventions [20,21]. Shortcomings of psychological modalities include stigma, in which case, some older adults may be reluctant to seek mental health support, and availability, where access to trained professionals and psychological services can be limited in some areas [19-22].

Physical Interventions

Strengths of physical interventions include (1) community-based exercise programs - these promote physical health and social interaction, as well as quality of life, and social engagement, with group participation and structured programs further promoting the effectiveness of these programs [23,24]; (2) group physical activities - these enhance social bonds provide physical benefits, and lead to reduced anxiety and loneliness [25]. Combining physical exercise with social interaction maximizes benefits [25]. A major limitation of physical interventions is mobility issues. Older adults with significant mobility limitations may find it difficult to participate. Another is remote accessibility. In this case, while tele-exercise offers alternatives, it may not be as effective as in-person interactions for some individuals [23-27].

Comparative synthesis of interventions

Effectiveness Across Interventions

Social interventions are highly effective in fostering direct social interactions and creating a sense of community and are particularly beneficial when they are culturally tailored and involve regular, structured engagement [5-11]. Technological interventions offer scalable and flexible solutions that can reach a broad audience, with their effectiveness hinging on ease of use, accessibility, and engaging content [12-18]. Psychological interventions address the underlying psychological aspects of loneliness, with structured approaches like CBT showing strong evidence of effectiveness [19-22]. Tailored psychotherapeutic and resilience-focused interventions also provide significant benefits [19-22]. Physical interventions combine physical health benefits with social engagement and are most effective when they include group activities and are tailored to the physical capabilities of participants [23-27].

Integration and Best Practices

Combination approaches: Integrating multiple types of interventions can provide a more comprehensive strategy for addressing SSS [28,29]. For example, combining social support with physical activities or integrating psychological elements into technological platforms [12].

Personalization: Tailoring interventions to meet the individual needs and preferences of older adults enhances their effectiveness, and includes considering cultural, socio-economic, and technological factors [5,7,8,13].

Support and training: Providing support and training for older adults to use technological interventions and encouraging participation in physical activities can improve outcomes [16,19-21].

Long-term sustainability: Ensuring the long-term sustainability of interventions through continuous funding, community support, and regular evaluation is crucial for maintaining their impact [3,30,31].

In summary, a comparative analysis and synthesis of interventions demonstrates that each type has distinct strengths in tackling SSS. Social interventions provide direct social engagement, technological interventions offer adaptable and scalable solutions, psychological interventions address psychological needs, and physical interventions combine health benefits with social interaction. By integrating these approaches and tailoring them to individual needs, a comprehensive strategy can effectively reduce loneliness and enhance the well-being of older adults [3]. Continued research and development are crucial to optimize these interventions, guarantee equitable access, and integrate them into holistic care strategies for the elderly [3].

Policy implications

Integration of Multidimensional Interventions

Holistic approach: To effectively address the multifaceted issue of loneliness among older adults, policies should integrate social, technological, psychological, and physical interventions [28,29,32]. Additionally, cross-sector collaboration is indispensable, and policies should encourage partnerships between primary care, specialized healthcare, and community services [3]. By amalgamating these approaches, comprehensive support can be provided, addressing the physical and psychological dimensions of loneliness [3].

Funding and resources: Governments and healthcare organizations should allocate adequate funding and resources to develop and sustain multidimensional intervention programs, and investments should be made in community centers, digital infrastructure, and training programs for professionals working with older adults [32].

Accessibility and Equity

Addressing the digital divide: Governments should invest in bridging the digital divide by offering affordable internet access and digital literacy training for older adults, as equitable access to technological interventions is crucial for reaching a broader audience [33,34].

Ensuring accessibility: Policies must guarantee that interventions are accessible to all older adults, including those with mobility issues, limited digital literacy, and socio-economic constraints [33,34]. This includes providing transportation to community centers, offering affordable or free digital devices, and ensuring programs are available in multiple languages and culturally tailored [33,34].

Support for Caregivers and Professionals

Training programs: Policies should mandate comprehensive training programs for caregivers and professionals to ensure the effective implementation and management of interventions [3]. This training should cover digital literacy, therapeutic techniques, and community engagement strategies [3,32].

Workforce development: Investing in the development of a skilled workforce that can support the mental, physical, and social health of older adults is crucial, and includes hiring and retaining professionals in geriatric care, social work, and mental health services [33,35].

Practical recommendations for implementing effective interventions

Community and Group-Based Programs

Regular and structured activities: Organization of regular and structured community activities that encourage social interaction, like weekly social gatherings, group exercise classes, and cultural events [36].

Cultural relevance: Programs should be tailored to meet the cultural and linguistic needs of diverse elderly populations [36]. Engage community leaders to ensure programs are culturally appropriate and well-received [36].

Leveraging Technology

User-friendly platforms: Develop and promote the use of user-friendly digital platforms that facilitate social interaction and mental health support, and ensure that these platforms are designed with older adults in mind, with intuitive interfaces and clear instructions [3].

Digital literacy training: Offer digital literacy training programs to help older adults become comfortable with using technology, and provide one-on-one support and ongoing assistance to address any technological challenges they may face [37].

Psychological Interventions

Group and individual therapy: Implement both group and individual psychological sessions, such as CBT and resilience training, to address the psychological aspects of loneliness, and ensure these services are readily available and covered by health insurance plans [3].

Telehealth services: Expand access to telehealth services, including teletherapy and online support groups, to reach homebound older adults and those in remote areas, and promote awareness of these services and guide on accessing them [17].

Physical Activity Programs

Accessible exercise programs: Create exercise programs suited to the physical abilities of older adults, including offering low-impact activities such as yoga, walking groups, and tai chi [36].

Combining social and physical activities: Integrate social components into physical activity programs to foster social engagement [36]. For example, organize group walks or dance classes that encourage interaction and community building [36].

Support and Training for Caregivers

Caregiver education: Develop comprehensive educational programs for caregivers to enhance their ability to support the social, emotional, and physical needs of older adults [3,32]. These programs should include training on effective communication techniques, mental health first aid, and methods for facilitating social activities [3,32].

Respite care services: Implement respite care services to provide caregivers with necessary breaks, helping to prevent burnout [33,35,37]. These services enable caregivers to maintain their well-being while ensuring that older adults continue to receive consistent, high-quality care [33,35,37].

Monitoring and Evaluation

Regular assessments: Implement regular assessments to monitor the effectiveness of interventions [36]. Use standardized tools to measure outcomes such as loneliness, social connectedness, and quality of life [36].

Feedback mechanisms: Establish feedback mechanisms to gather input from participants and caregivers, and to continuously improve and adapt programs to better meet the needs of older adults [37].

Summary of reviewed studies

Table 1 summarizes the reviewed studies.

**Table 1:** Summary of reviewed studies CBT: cognitive-behavioral therapy; ICT: information and communication technology; IL: interleukin

Author(s)	Year	Study Design	Sample Size	Location	Intervention	Outcome Measures	Key Findings
Lai et al. [5]	2020	Randomized Controlled Trial	60	Canada	Peer-based intervention for older Chinese immigrants	Loneliness, Social Isolation	Effective in reducing loneliness and social isolation among older Chinese immigrants.
Rodríguez-Romero et al. [6]	2021	Randomized Controlled Trial	55	Spain	Social intervention to reduce perceived loneliness	Perceived loneliness	Effective in reducing perceived loneliness among community-dwelling older adults
Warner et al. [7]	2024	Dual Randomized Controlled Trial	375	Hong Kong	Volunteering	Loneliness, social and mental health	Volunteering significantly reduced loneliness and improved mental health outcomes.
Galinha et al. [8]	2022	Randomized Controlled Trial	149	Portugal	Singing group program	Subjective and social well-being	Enhanced well-being and reduced loneliness through group singing.
Watson et al. [9]	2024	Randomized Controlled Trial	130	Canada	Creative aging programs	Healthy aging	Creative activities promoted healthy aging and improved quality of life.
Conwell et al. [10]	2021	Randomized Controlled Trial	427	USA	Peer companionship	Mental health outcomes	Improved mental health outcomes in older adults through peer companionship.
Qorbani et al. [11]	2024	Randomized Controlled Trial	80	Iran	Support groups	Retirement syndrome	Reduced symptoms of retirement syndrome in older adults
Yang et al. [12]	2023	Pilot study followed by a randomized controlled trial.	89	Taiwan	Online interactive courses designed to reduce loneliness and improve the quality of life among older adults during the COVID-19 pandemic.	Loneliness and quality of life	The study found that online interactive courses were effective in reducing loneliness and improving the quality of life of older adults during the pandemic, suggesting the viability of online platforms in delivering therapeutic content.
Tsai et al. [13]	2020	Quasi-experimental study	62	Taiwan	Smartphone-based videoconferencing	Depression, loneliness, quality of life	Reduced depression and loneliness; improved quality of life for nursing home residents.
Jarvis et al. [14]	2019	Randomized Controlled Trial	828	South Africa	mHealth-supported low-intensity CBT	Loneliness	Reduced loneliness in older people through mHealth-supported CBT.
Ali et al. [15]	2021	Randomized Controlled Trial	20	USA	Online conversational skills coach	Social engagement	Improved social engagement and reduced loneliness with conversational skills coaching.
Choi et al. [16]	2020	Randomized Controlled Trial	89	USA	Tele-delivered behavioral activation vs. tele-delivered friendly visits	Social connectedness	Tele-delivered behavioral activation improved social connectedness compared to friendly visits.
Bruce et al. [17]	2021	Randomized Controlled Trial	64	USA	Tele-delivered behavioral activation vs. tele-delivered friendly visits	Social connectedness (1-year impact)	One year later, tele-delivered behavioral activation sustained improved social connectedness.
Funghi et al. [18]	2024	Randomized Controlled Trial	60	Italy	Socio-Cognitive Online Training Project	Socio-cognitive well-being	Improved socio-cognitive well-being in older adults through digital training.
Smith et al. [19]	2021	Randomized Controlled Trial	62	Australia	Group Cognitive Behavioral Therapy	Loneliness	Significant reduction in loneliness through group CBT.
Jesus et al. [20]	2024	Randomized Controlled Trial	199	Portugal	Homebound elderly people psychotherapeutic intervention (HEPPI)	Mediating role of loneliness in the effectiveness of the psychotherapeutic intervention.	The intervention explored how psychotherapeutic strategies could mediate loneliness among homebound elderly individuals, potentially leading to improvements in their overall psychological well-being.
Jeste et al. [21]	2023	Pilot Controlled Clinical Trial	20	USA	Resilience- and Wisdom-Focused Intervention	Stress, Loneliness	Reduced perceived stress and loneliness in older adults.
Solomonov et al. [22]	2019	Behavioral Activation Therapy	48	USA	Social and interpersonally rewarding activities	Depression outcomes	Engagement in rewarding activities predicted better outcomes in late-life depression.
Olsen et al. [23]	2022	Randomized Controlled Trial	215	Denmark	Community-based exercise	Independent living	Enhanced independent living through community-based exercise with self-management.
Zengin Alpozgen et al. [24]	2022	Randomized Controlled Study	30	Turkey	Synchronous tele-exercise	Physical Fitness, Quality of Life, Mood	Maintained physical fitness, quality of life, and mood in older people.
Liu et al. [25]	2024	Randomized Controlled Trial	38	China	Reminiscence Therapy-Based Board Game	Anxiety, loneliness	Decreased anxiety and loneliness levels through a hybrid board game.
Larsen et al. [26]	2020	Randomized Controlled Trial	Not specified	Denmark	Motivational interviewing, physical activity monitoring	Physical activity, quality of life, loneliness	Prospective study
Ristolainen et al. [27]	2020	Randomized Controlled Trial	392	Finland	Group-based care management	Well-being	Improved well-being in older people living alone through participatory care management.
Ozturk et al. [38]	2023	Randomized Controlled Trial	65	Turkey	Social intervention aimed at reducing perceived loneliness among community-dwelling older people	Perceived loneliness	The intervention was effective in reducing perceived loneliness in community-dwelling older adults, highlighting the importance of targeted social interventions to enhance the social well-being of this population.
Borji and Tarjoman [39]	2020	Randomized Controlled Trial	88	Iran	Religious intervention aimed at enhancing mental vitality and reducing the sense of loneliness among the elderly attending community healthcare centers.	Mental vitality and sense of loneliness.	The religious intervention was effective in increasing mental vitality and decreasing feelings of loneliness among the elderly, emphasizing the potential therapeutic benefits of spiritual activities
Xie et al. [40]	2019	Randomized Controlled Trial	80	China	Modified behavioral activation treatment for depression	Depressive symptoms	Effective in reducing depressive symptoms in rural left-behind elderly.
Alici and Bahceli [41]	2021	Randomized Controlled Trial	62	Turkey	Laughter Therapy	Life satisfaction, loneliness	Improved life satisfaction and reduced loneliness in nursing home residents.
Mächler et al. [42]	2023	Randomized Controlled Trial	29	Germany	Social activities intervention in general practices	Patient experiences, social activities	Positive patient experiences; enhanced social activities among older patients.
Li et al. [43]	2022	Randomized Controlled Trial	64	China	Group reminiscence therapy	Loneliness, stress	Reduced loneliness and perceived stress through reminiscence therapy.
Ae-Ri et al. [44]	2023	Randomized Controlled Trial	40	South Korea	ICT-based Loneliness Alleviation Program	Loneliness	Effective in reducing loneliness in community-dwelling older adults.
Shapira et al. [45]	2021	Randomized Controlled Trial	82	Israel	Web-based CBT and mindfulness skills	Mental health	Improved mental health during COVID-19 via web-based interventions.
Hernadez-Ascanio et al. [46]	2023	Randomized Controlled Trial	119	Spain	Multicomponent intervention to reduce social isolation and loneliness	Social isolation, loneliness	The intervention significantly reduced social isolation and loneliness in community-dwelling elders.
Ofosu et al. [47]	2023	Randomized Controlled Trial	49	Scotland	Digital music and movement intervention	Feasibility, acceptance	Feasible and well
Chow et al. [48]	2019	Randomized Controlled Trial	125	China	Dual-Process Bereavement Group Intervention	Bereavement, Loneliness	Reduced bereavement and loneliness in widowed older adults
Gilbody et al. [49]	2021	Randomized Controlled Trial	96	England	Behavioural activation for depression and loneliness	Depression, Loneliness	Prevented depression and reduced loneliness among older people with long-term conditions.
Boekhout et al. [50]	2021	Randomized Controlled Trial	585	Netherlands	Computer-tailored intervention for older adults with chronic diseases	Loneliness	Long-term reduction in loneliness through a computer-tailored intervention.
Ohashi and Katsura [51]	2019	Randomized Controlled Trial	Not specified	Japan	Coaching program	Quality of life	Improved quality of life in older Japanese adults through a structured coaching program.
Johnson et al. [52]	2020	Randomized Controlled Trial	390	USA	Community choir	Well-being	Community choir participation promoted well-being and social engagement among diverse older adults.
Lindsay et al. [53]	2022	Randomized Controlled Trial	190	USA	Mindfulness-based stress reduction	IL-6 production, loneliness	Increased IL-6 production; reduced loneliness among lonely older adults.
Brodbeck et al. [54]	2019	Randomized Controlled Trial	110	Germany	Guided internet-based self-help intervention	Bereavement, separation/divorce outcomes	Reduced symptoms of bereavement and separation/divorce through self-help intervention.

## Conclusions

In conclusion, the review of various interventions for SSS highlighted several key insights. Each type of intervention - social, technological, psychological, and physical - has unique strengths in addressing the multifaceted nature of loneliness and enhancing the quality of life for older adults. The effectiveness of a multidimensional approach is particularly emphasized. Combining social, technological, psychological, and physical interventions can create a holistic strategy to combat isolation and improve the overall well-being of older adults.

Indeed, addressing SSS is crucial not only for mental and physical health but also for enhancing the quality of life and mitigating the effects of this syndrome. Continued research, policy support, and practical implementation of these interventions are essential to creating inclusive and supportive communities for our aging population.
